# To Feed or Not to Feed: Plant Factors Located in the Epidermis, Mesophyll, and Sieve Elements Influence Pea Aphid’s Ability to Feed on Legume Species

**DOI:** 10.1371/journal.pone.0075298

**Published:** 2013-09-30

**Authors:** Alexander Schwarzkopf, Daniel Rosenberger, Martin Niebergall, Jonathan Gershenzon, Grit Kunert

**Affiliations:** Department of Biochemistry, Max Planck Institute for Chemical Ecology, Jena, Germany; INRA-UPMC, France

## Abstract

The pea aphid (*Acyrthosiphon pisum* Harris), a legume specialist, encompasses at least 11 genetically distinct sympatric host races. Each host race shows a preference for a certain legume species. Six pea aphid clones from three host races were used to localize plant factors influencing aphid probing and feeding behavior on four legume species. Aphid performance was tested by measuring survival and growth. The location of plant factors influencing aphid probing and feeding was determined using the electrical penetration graph (EPG) technique. Every aphid clone performed best on the plant species from which it was originally collected, as well as on *Vicia faba.* On other plant species, clones showed intermediate or poor performance. The most important plant factors influencing aphid probing and feeding behavior were localized in the epidermis and sieve elements. Repetitive puncturing of sieve elements might be relevant for establishing phloem feeding, since feeding periods appear nearly exclusively after these repetitive sieve element punctures. A combination of plant factors influences the behavior of pea aphid host races on different legume species and likely contributes to the maintenance of these races.

## Introduction

The pea aphid (*Acyrthosiphon pisum* Harris) is confined to plants of the family Fabaceae. Within the last 6500–9500 years, this aphid underwent a rapid genetic diversification involving host plant shifts [1], probably influenced by global warming and anthropogenic range expansion of potential hosts. As a consequence, pea aphid populations now occur sympatrically on legume crop plants [2] as well as on legume species in natural habitats [3]. Pea aphid populations are often very specialized, performing best on the particular legume species on which they are found (called native host plant), but showing significantly reduced performance, or not surviving at all on other legumes [4], [5]. However, all pea aphid populations tested so far perform as well on *Vicia faba* as on their native host plant. Thus, *V. faba* can be considered as a “universal host plant” for the genetically diverse populations of this species [4], [5]. By investigating more than 1000 wingless pea aphids from 19 legume species in western Europe, Peccoud et al. [3] identified 11 genetically distinct and sympatrically occurring pea aphid races associated with different legume host plants. Analysis of migration and hybridization among these races led to delineation of three possible species and eight host races.

The distinct plant preferences of pea aphid host races lead to assortative mating which reduces gene-flow [2], [6]. On the other hand the presence of *V. faba* as a universal host allows the different races to meet and mate [5]. The presence of ongoing gene flow amongst host races raises the question of how the host races are maintained. Plant factors are very likely to be involved as aphid feeding behavior involves an intimate relationship with its host. Numerous studies on different plant-aphid systems have shown that a range of plant factors influence the plant-aphid interaction (reviewed in [7]–[9]). Relevant plant factors differ among various plant-aphid combinations and can function at different stages of host selection as aphids land on the plant, penetrate tissues with their stylets and establish feeding sites in phloem. For example, plant factors that influence aphid host selection can be located at the plant surface in the form of attractive [10] or repellent volatiles [11], deterrent epicuticular lipids [12] or glandular trichomes [13]. Such factors can also be located elsewhere in the plant including deterrent gustatory cues in the epidermis [14], or compounds inhibiting stylet penetration in the mesophyll [13]. In sieve elements (SEs), phloem sap may have low nutritional value for the aphid [15], and barriers that prevent the aphid from starting to feed [13], [16]–[19]. In addition to attractive or deterrent plant factors, different nutrient levels or the presence or absence of certain compounds may also influence aphid host selection [20]. The variety of such factors and their distinct mode of action in different plant species may have been critical in driving aphid speciation. Thus to understand the diversification among pea aphid lineages, the nature of factors affecting host selection among closely related aphid taxa must be better investigated. The results should be applicable to other polymorphic aphid species or species complexes that feed on an assortment of different plant species.

**Figure 1:**
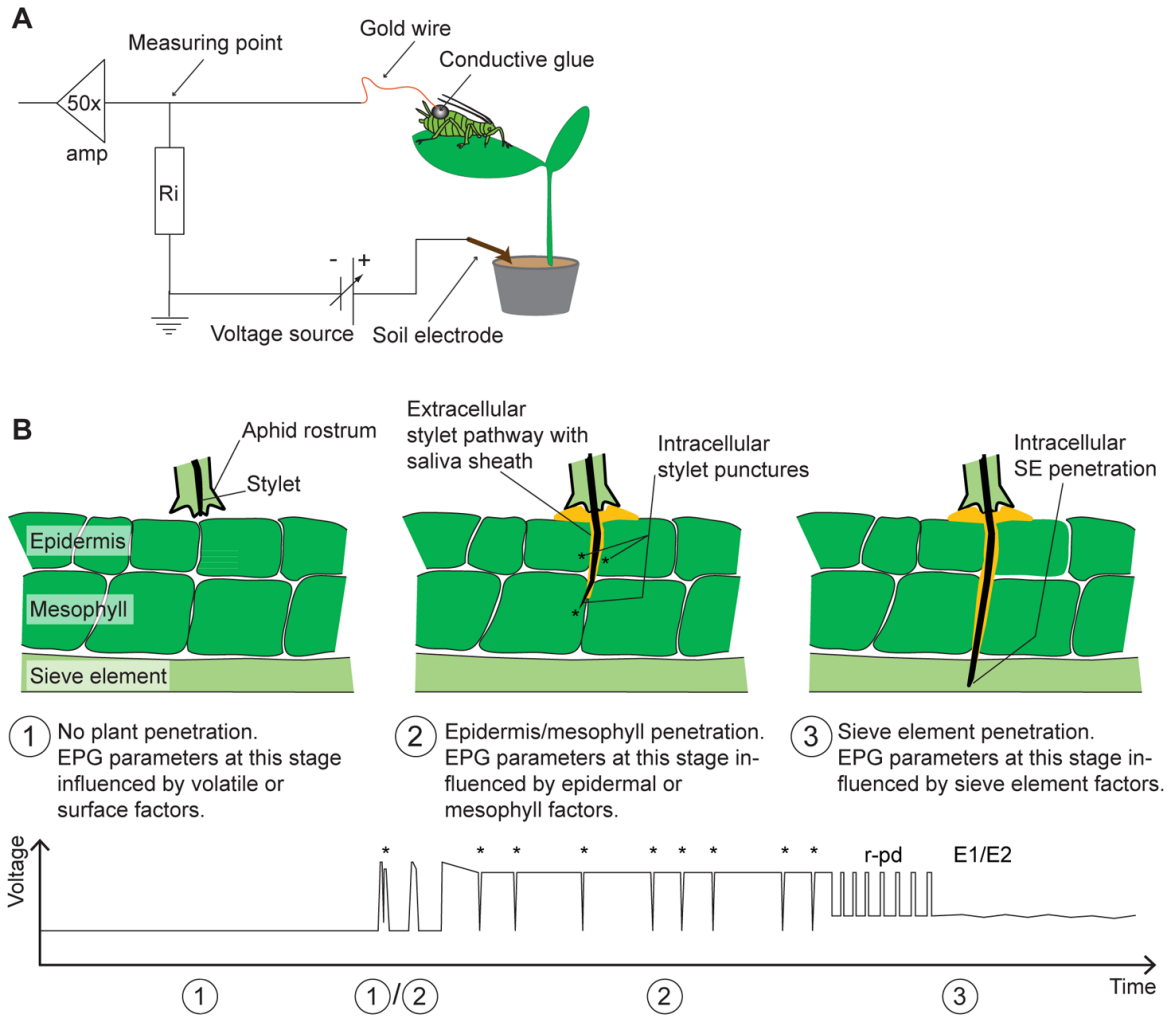
Principle of EPG technique and plant factor localization modified after Tjallingii [53]. A) Principle of EPG technique: the aphid is connected to the EPG device using conductive glue and a thin gold wire. The plant is connected to the EPG device by inserting an electrode into the soil next to the plant. As soon as the aphid starts plant penetration the electrical circuit is closed and EPG waveforms can be observed and recorded. Amp = amplifier, Ri = input resistor. B) Simplified illustration of various stages in the penetration of plant tissue by the aphid stylets correlated with a schematic representation of EPG recordings below. The parameters derived from the EPG recordings (listed in Table S1) indicate the tissue location of plant factors influencing stylet penetration and feeding behavior. (1) As long as the aphid does not penetrate the plant a flat line, called non-probing waveform (np) is visible. EPG parameters from this stage are influenced by volatile or surface plant factors influencing aphid feeding behavior (Table S1, parameters #1–#4). (1)/(2) When the aphid starts penetration, short probes can often be observed, some with cell punctures (potential drops (pd) marked by asterisks) and separated by non-probing periods. The number and duration of short probes are influenced by factors in the epidermis (parameter #5) or the mesophyll (#8). (2) During the pathway phase (#15) the aphid navigates its stylet bundle through the plant apoplast towards the sieve elements (SEs). Almost every single cell along the stylet pathway is punctured (pd, marked by asterisks) by the aphid’s stylet (#16). Aphid activities during epidermis and mesophyll penetration are reflected in parameters #5–#24. (3) Parameters #25–#51 reflect SE factors, important as the SEs are the aphid’s ultimate feeding target. Before SE salivation (E1) and ingestion (E2), *A. pisum* often carries out extended and repetitive cell punctures (r-pd).

An excellent method to investigate plant-aphid interactions and to localize plant factors that influence these interactions is the electrical penetration graph (EPG) technique [13], [14], [16]–[19], [21], [22] (Figure 1) which monitors aphid probing and feeding behavior in detail. By comparing numerous parameters of the probing and feeding behavior of aphid individuals on resistant and susceptible plant species it is possible to detect and locate plant factors influencing plant resistance or susceptibility. This approach has also been used to detect and localize plant factors influencing the host range of pea aphids on different legume species [22]–[24]. Whilst Wilkinson and Douglas [23] investigated mainly interclonal differences in the probing and feeding behavior of pea aphid clones from the Medicago, Pisum and Trifolium races on *Pisum sativum* and the universal host plant *V. faba*, other authors (Caillaud [24]; Caillaud and Via [22]) focused on susceptible and resistant plants by using clones from the Medicago and Trifolium races on *Medicago sativa* and *Trifolium pratense*. However, to more fully account for plant specific factors a broader overview of different host plants in combination with different aphid clones from multiple host races is needed.

A previous study on two pea aphid races revealed that individuals are able to recognize the suitability of a potential host plant by briefly puncturing epidermal or subepidermal plant cells [22]. Later it was shown by Del Campo et al. that this behavior depends on recognition of chemical substances from the native host plant [25]. Pea aphid clones with either *M. sativa* or *T. pratense* as native hosts were able to recognize stimulants in the extract from their respective native host. Although both studies [22], [25] made important advances in understanding pea aphid host plant use, many questions remain unsolved, such as where the recognized chemical substances of Del Campo et al. [25] are located. Additionally, beyond simple host vs. non-host recognition [22], pea aphid races can accept plants other than their native or universal host plant and show an intermediate performance [3]. Feeding of aphids on such intermediate hosts might facilitate hybridization among host races and act against speciation.

Plant factors influencing aphid performance after initial host/non-host choices have been made are more likely to be in deeper plant layers including the phloem. Previous studies demonstrated that features of the sieve elements (SEs) themselves were responsible for reduced feeding in various plant-aphid associations: in the pea aphid and in *Acyrthosiphon kondoi* on *Medicago truncatula* [19], [26], in *Macrosiphum euphorbiae* on *Solanum lycopersicum* [17], [27], and in *Aphis gossypi* on *Cucumis melo* [28]. In these cases, plant resistance genes (*R*-genes) were found to be involved in SE-specific aphid resistance. *R*-genes mediate the recognition of effector proteins delivered by the aphid saliva to the plant (reviewed in [29]). On the other hand, aphid saliva effector molecules suppressing plant defense responses that are specific to aphid and host plant species were also characterized [30]–[33]. However, it remains unclear if plant *R*-genes and aphid salivary effectors play a role in determining the acceptance of host plants to the various pea aphid host races.

Our study focuses on the detection and localization of plant factors influencing probing and feeding behavior of various pea aphid host races on different legume species. These plant factors may contribute to the maintenance of host races in the pea aphid species complex. As it is crucial to know the performance of each clone on each plant species, we firstly characterized the performance (survival, growth) of six pea aphid clones belonging to three races on four legume species, including the native host plant, the universal host-plant *V. faba* and non-host plants. Secondly, we performed EPG recordings for each aphid clone-plant combination to localize putative plant factors responsible for differential performance.

## Materials and Methods

### Plants

Four different legume species were used in in this study: *Medicago sativa* cv. “Giulia” (Appels Wilde Samen GmbH, Darmstadt, Germany), *Pisum sativum* cv. “Baccara” (S.A.S. Florimond Desprez, Cappelle-en-Pévèle, France), *Trifolium pratense* cv. “Dajana” (Appels Wilde Samen GmbH, Darmstadt, Germany), and *Vicia faba* cv. “The Sutton” (Nickerson-Zwaan, Made, The Netherlands). All plants were reared in 10 cm diameter pots on plant substrate “Klasmann Tonsubstrat” (Klasmann-Deilmann GmbH, Geeste, Germany) in a climate chamber under the following conditions: 20°C, 70% relative humidity, 16 hours light per day.

### Aphids

Six different *Acyrthosiphon pisum* Harris clones occurring sympatrically in Western Europe were used. The aphid clones were collected in the field from three legume species: clones “L1_22” and “L84” (called M1 and M2) from *M. sativa*; clones “P136” and “Colmar” (P1 and P2) from *P. sativum*; clones “YR2” and “T3_8V1” (T1 and T2) from *T. pratense* (for detailed clone information see Table S1 in [1]). All aphid clones were maintained on *V. faba* cv. “The Sutton” covered with air-permeable cellophane bags (Armin Zeller, Nachf. Schütz & Co, Langenthal, Switzerland) to prevent aphid cross-contamination. Conditions for all aphid rearing in this study were: 20°C, 70% relative humidity, 16 hours light per day.

For each experiment aphid clones were reared on *V. faba* starting from one apterous adult aphid which was placed on a *V. faba* plant and allowed to reproduce for two days. After two days, the adult aphid was removed and larvae kept on the plant until adulthood. These adult aphids were transferred to new *V. faba* plants (one aphid per plant). This rearing process was repeated several times until a sufficient number of aphids for the experiments were obtained.

### Aphid Performance

Each aphid clone listed in the previous section was tested on the four legume species: *M. sativa*, *P. sativum*, *T. pratense*, and *V. faba*. At the beginning of the experiment ten first-instar larvae from each clone were placed on the soil close to the base of a 27 day-old plant. Each aphid clone-plant combination was replicated five times and set up in a spatially randomized pattern in a climate chamber. After nine days, all surviving individuals per plant were counted and weighed, and the average weight per surviving individual was calculated. Statistical analysis was performed by using R version 2.12.2 [34]. The effect of plant species, aphid clone and plant species-aphid clone interaction on the number of surviving aphids was tested using generalized linear models with a poisson/quasipoisson error family. The effect of plant species, aphid clone and plant species-aphid clone interaction on aphid survivor weight was tested with a two-factorial ANOVA.

### Monitoring Aphid Probing and Feeding Behavior by the EPG Technique

Each aphid clone was tested on the four legume species: *M. sativa*, *P. sativum*, *T. pratense*, and *V. faba*. For each EPG recording, a 9–11 day old adult aphid was immobilized on a disposable pipette tip connected to a vacuum pump. A small droplet of conductive silver-glue (EPG Systems, Wageningen, The Netherlands) was applied to the aphid’s dorsum. The tip of a 2 cm long gold-wire (diameter 20 µm) connected to an insect electrode (prepared from a 1.5 cm long copper pin) was inserted into the glue droplet. The wired aphid was placed on a 27–32 day-old experimental plant at the edge of the adaxial side of the uppermost fully developed leaf, which was fixed by a hair-clip. The soil electrode was inserted into the soil. This procedure was repeated eight times to equip each of the eight EPG probes of the direct current-EPG device (“GIGA-8”, EPG Systems, Wageningen, The Netherlands). The experimental plants and the EPG device equipped with aphids were then placed in a Faraday cage. The EPG device was connected via an USB analog-digital converter device (“DI 710”, DATAQ Instruments, Akron OH, USA) to a computer. As the aphid starts penetrating the plant by inserting its stylet bundle into the plant tissue, the electrical circuit is closed and EPG waveforms (i.e. voltage changes over time) can be recorded (Figure 1). EPG recordings were conducted for 4 hours using the software “Probe 3.5” (EPG Systems, Wageningen, The Netherlands). For each aphid clone-plant combination, 17–24 4 h EPG recordings were conducted in which aphids were successful in initiating probing during the recording time. In preliminary experiments, a time of 4 h was found to be sufficient for nearly all of our experimental aphid clones to reach a sustained feeding phase on their native host plants. The exceptions, clones of the Medicago host race, are described in the discussion section.

The beginning and the end of each EPG waveform (Table S1, “EPG waveforms”; Figure 1) in all EPG recordings were marked manually using “Stylet a+” software (version v01.00 26.08.2010, EPG Systems, Wageningen, The Netherlands). Subsequently, 54 EPG parameters representing aphid probing and feeding behaviors were calculated (Table S1, “EPG parameter”) by using a Microsoft Office Excel Macro designed for our purposes. It calculates a wide range of standard EPG parameters like other Macros available for EPG data processing (e.g. [35]), but also calculates the number and total duration of repetitive SE puncture periods (r-pd) and their association with SE salivation (E1), SE feeding (E2) and sustained (longer than 10 min) SE feeding (Table S1, #41–#51). Statistical analysis was performed by using R version 2.12.2 [34]. The effect of each plant species on each of the 54 EPG parameters was tested for every clone separately. If an EPG parameter was observed in less than five replicates in an aphid clone-plant combination, the respective combination was excluded from the analysis. The effect of plant species on the proportion of individuals showing a certain EPG parameter was tested by using the test for equality of proportions. Plant effects on the total time an aphid clone spent in a certain waveform during 4 h recording time were tested using one-factorial ANOVA (after appropriate data transformation, if necessary). In case of non-normality of the errors or inequality of variances, the non-parametric Kruskal-Wallis-Test was applied. Plant effects on the value of EPG parameters for each aphid clone during the 4 h recording time were tested by using generalized linear models with a poisson/quasipoisson error family. For full information about test statistics for each parameter and applied transformations please refer to Table S3. Plant effects on the average number of repetitive SE puncture periods with and without subsequent SE feeding phases and on the average number of SE feeding phases with and without preceding repetitive SE puncture periods were tested using generalized linear models with a binomial/quasibinomial error family (Table 3, Table S2).

## Results

### Aphid Performance

#### Survival

**Figure 2:**
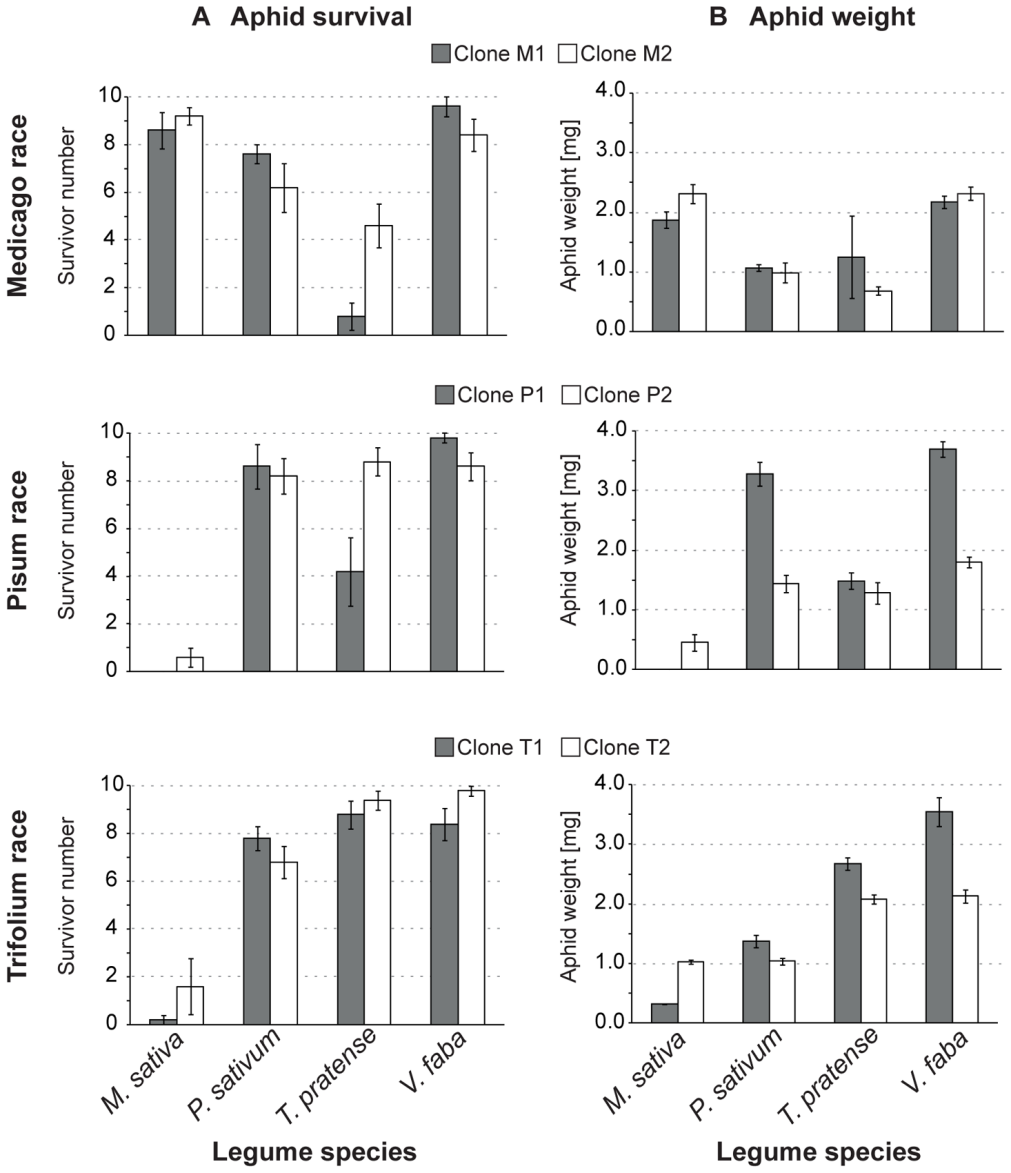
Performance of each clone of the pea aphid host races on the four legume species. Performance was best on the plant the aphid clones were originally collected from (those plants called native host plants). On *V. faba* all aphid clones were able to perform well (universal host plant). On other plants, aphid clones were able to perform intermediately (less-suitable plants) or were not able to survive at all (non-host plants). Bars represent mean +/− Std. Error. Number of aphid nymphs at start of experiment was 10. Each treatment was replicated five times. A) Survivor number after nine days. B) Aphid weight [mg] after nine days. For test statistics, see Table 1.

Aphid survival for each clone was assessed on *M. sativa*, *P. sativum*, *T. pratense*, and *V. faba*. When the performance of the various aphid clones was compared on the four legume species, nearly all of the aphids survived on their native host plants. This was also true for aphids on the universal host plant *V. faba* regardless of host race or clone (Figure 2 A, Table 1). On other plant species normally not used as hosts, aphid survivor numbers were significantly lower than on the native or universal host plant, as for the Pisum and Trifolium race clones on *M. sativa* plants. These plant species can be considered non-hosts for the respective aphid clones. Besides native and universal host plants on the one hand and non-host plants on the other, there is a third group of plant species on which aphid survival is essentially as good as on host plants (like clone P2 on *T. pratense*) or in between the survival on host and non-hosts (like clone M2 on *P. sativum* and *T. pratense* or clone P1 on *T. pratense*). These plant species are designated as less suitable or intermediate hosts. Within an aphid host race, the number of survivors of each clone was similar on the universal and the native host plants, but on less suitable or non-host plants the number of survivors sometimes differed. For example within the Medicago race, clone M1 showed a significantly lower survivor number on *T. pratense* compared to clone M2. Within the Pisum race, survival of clone P1 on *T. pratense* was significantly lower than survival of clone P2, which survived on *T. pratense* as well as on the native and universal host plants.

**Table 1:** Test statistics on performance data of clones of aphid host races feeding on the various legume species.

	Medicago race		Pisum race		Trifolium race	
Survivor number	*F*	*P*	*F*	*P*	*F*	*P*
Plant	19.466	**<0.001**	30.268	**<0.001**	36.054	**<0.001**
Clone	0.805	0.376	2.690	0.110	1.638	0.209
Plant:Clone	5.244	**0.005**	4.883	**0.007**	2.478	0.079
**Average weight**						
Plant	50.690	**<0.001**	45.127	**<0.001**	70.568	**<0.001**
Clone	0.239	0.629	119.567	**<0.001**	58.771	**<0.001**
Plant:Clone	3.696	**0.023**	19.273	**<0.001**	10.342	**<0.001**

The influence of plant species and aphid clone identity on the survivor number was analyzed separately for the three aphid races using generalized linear models with a quasipoisson error structure. The influence of plant species and clone identity on the average aphid weight was analyzed separately for the three aphid races using two-factorial ANOVA. *P*-values below significance level (*P*<0.05) are printed in bold letters. Mean values and standard errors shown in Figure 2.

#### Survivor weight

In general, the weight of aphid survivors was at least twice as high on the native and universal host plants compared to aphid weight on less suitable or non-host plants (Figure 2 B, Table 1). However, the two clones of each host race did not always respond in the same way. Within the Pisum race, clone P2 showed nearly the same weight on *T. pratense* as on the native and universal host plants whereas clone P1 showed a significantly lower weight on *T. pratense*. Within the Trifolium race, clone T2 showed a significantly lower weight than clone T1 on the native and the universal host plant.

### Aphid Probing and Feeding Behavior

EPG recordings were conducted for each aphid clone on each of the four plant species to localize the factors important for aphid feeding (Figure 1 B). Parameters derived from analysis of EPG waveforms were used to assess aphid behavior in specific plant tissues (Table S1).

#### General parameters reflecting multiple tissue levels

**Figure 3:**
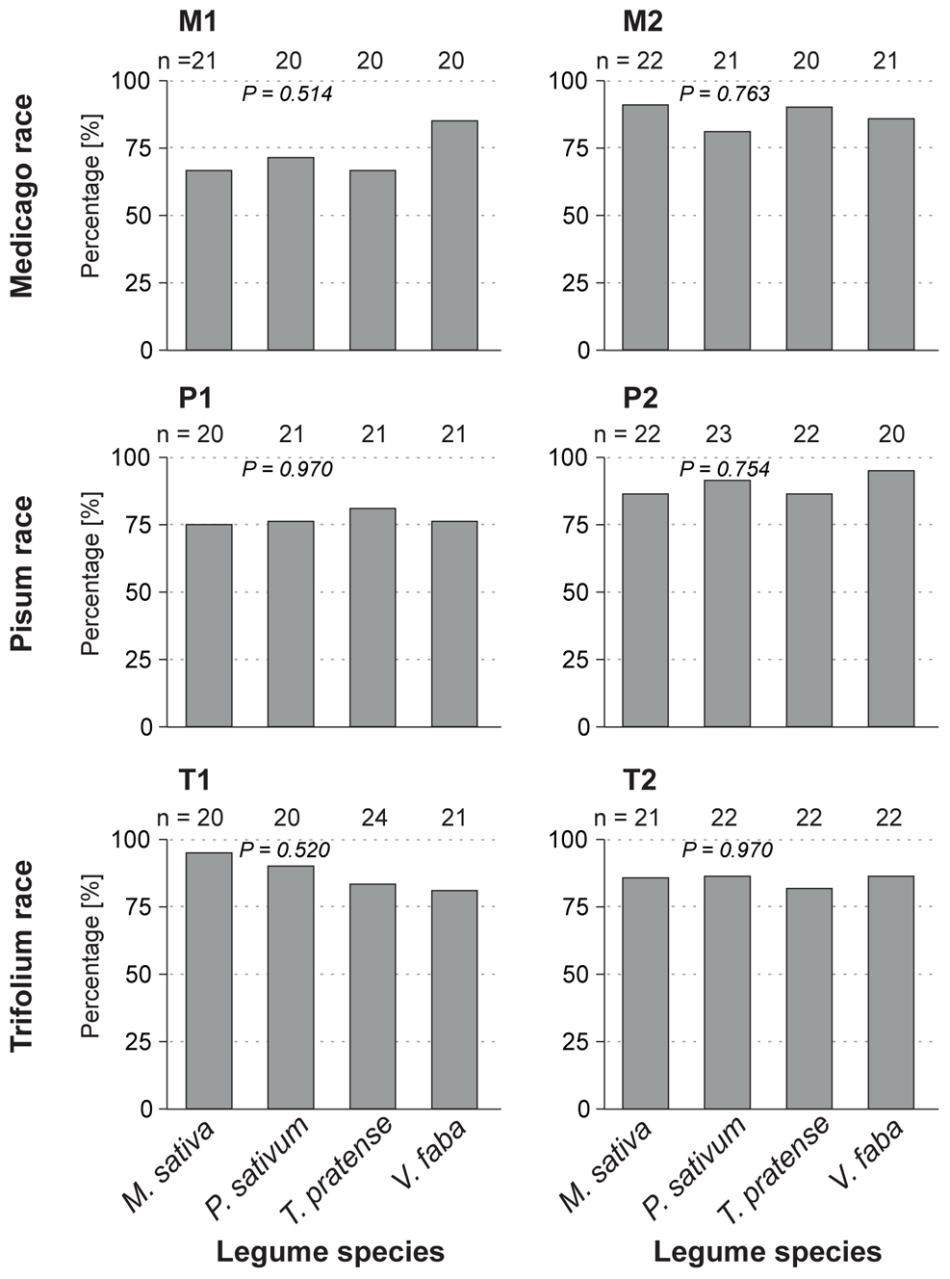
Percentage of aphid individuals initiating plant penetration. The graph shows the percentage of individuals for clones (M1, M2, P1, P2, T1 and T2) of each pea aphid host race initiating plant penetration (Table 2, parameter #1) on all four legume species throughout the entire EPG recording period. For details about test statistics, see Table S3.

During the 4 h recording time, 70–95% of aphid individuals from all clone-plant combinations started to penetrate the plant with their stylet (Figure 3). The identity of the plant species did not have any influence on the proportion of individuals starting stylet penetration (Table 2, Parameter #1). The total duration of stylet penetration of the aphid clones ranged between ∼2000 and ∼12000 s during the 4 h ( = 14400 s) EPG recording. But for clones P1, P2 and T1, the total stylet penetration times on native and universal host plants were significantly (two to four times) longer than on less-suitable or non-host plants (Table 2, #2).

**Table 2:** *P*-values of EPG parameters discussed in text.

			Medicago race		Pisum race		Trifolium race	
Tissue	#	EPG Parameter	M1	M2	P1	P2	T1	T2
Multiple	1	Proportion of individuals starting penetration	0.514	0.763	0.970	0.754	0.520	0.970
	2	Total penetration time	0.074	0.110	**<0.001**	**<0.001**	**<0.001**	0.071
Volatile/Surface	3	Time from start of experiment to first probe	0.710	**0.025**	**0.031**	0.167	0.145	0.501
Epidermis	5	Number of probes shorter than 30 s	0.659	**0.021**	0.651	0.278	**0.017**	**0.005**
	6	Number of probes shorter than 30 s without/with cell puncture	–	–	–	–	**0.008**	**<0.001**
Epidermis/Mesophyll	8	Number of probes shorter than 3 min	0.389	0.171	0.162	0.218	0.563	0.283
Mesophyll	15	Total duration of pathway phase	**0.001**	**<0.001**	**0.009**	0.138	**0.042**	**0.004**
Sieve elements	25	Proportion of individuals showing SE salivation	**<0.001**	**0.014**	**<0.001**	**<0.001**	**<0.001**	**<0.001**
	36	Proportion of individuals showing SE feeding	**<0.001**	**0.009**	**<0.001**	**<0.001**	**<0.001**	**<0.001**
	39	Proportion of individuals showing sustained SE feeding	**<0.001**	**0.003**	**<0.001**	**<0.001**	**<0.001**	**<0.001**
	41	Proportion of individuals showing repetitive SE punctures	**0.003**	**<0.001**	**0.002**	**0.003**	**<0.001**	**<0.001**

**Figure 4:**
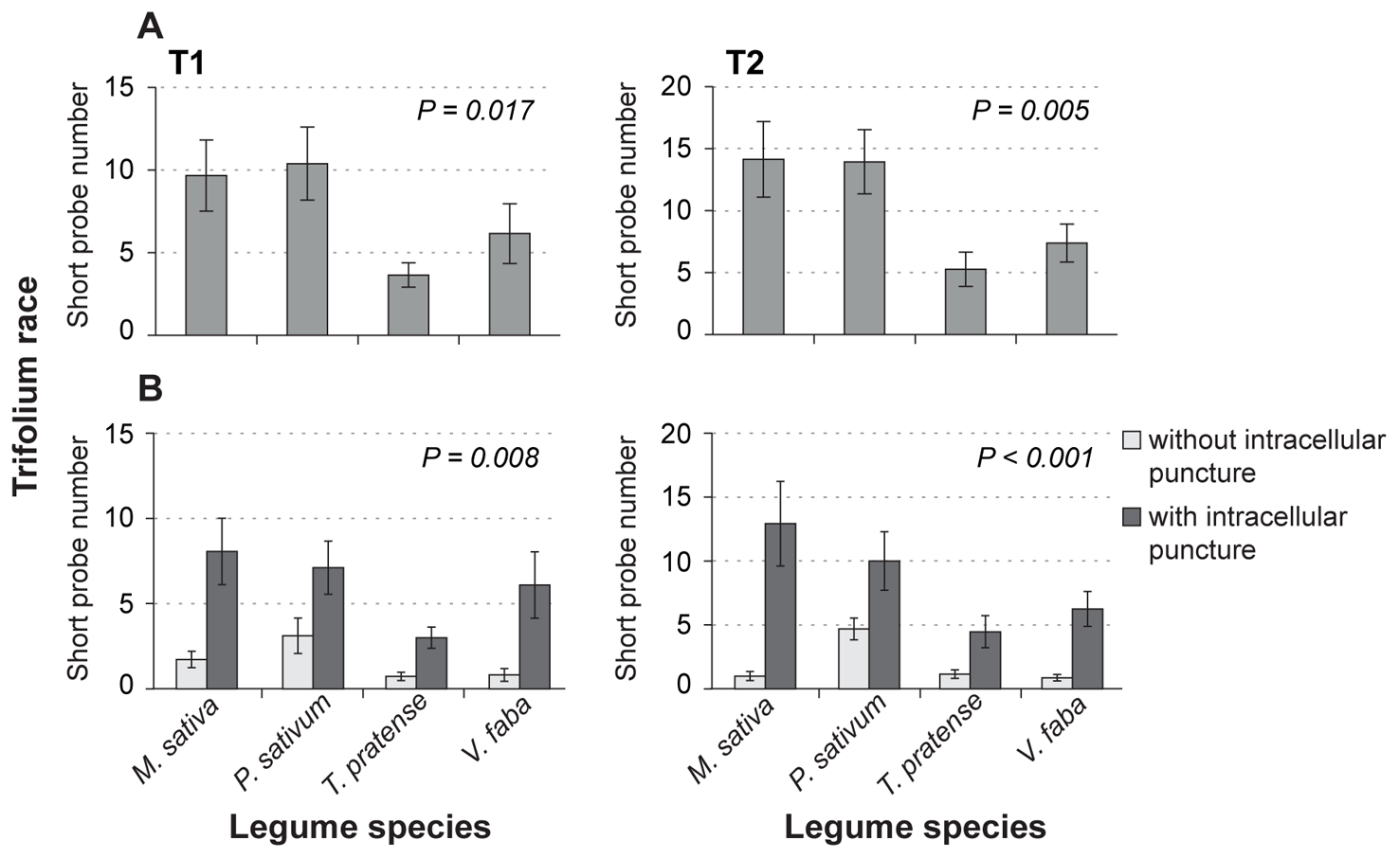
Number of very short probes (<30 s) during 4 h EPG recording. All bars represent mean numbers +/− standard errors. *n* = 14–21. A) The graph shows the numbers of very short probes (Table 2, parameter #5) made by two clones (T1, T2) of the Trifolium host race on the four legume species. B) The graph shows the proportion of very short probes without (light bars) and with (dark bars) intracellular punctures. For details about test statistics, see Table S3.

**Figure 5:**
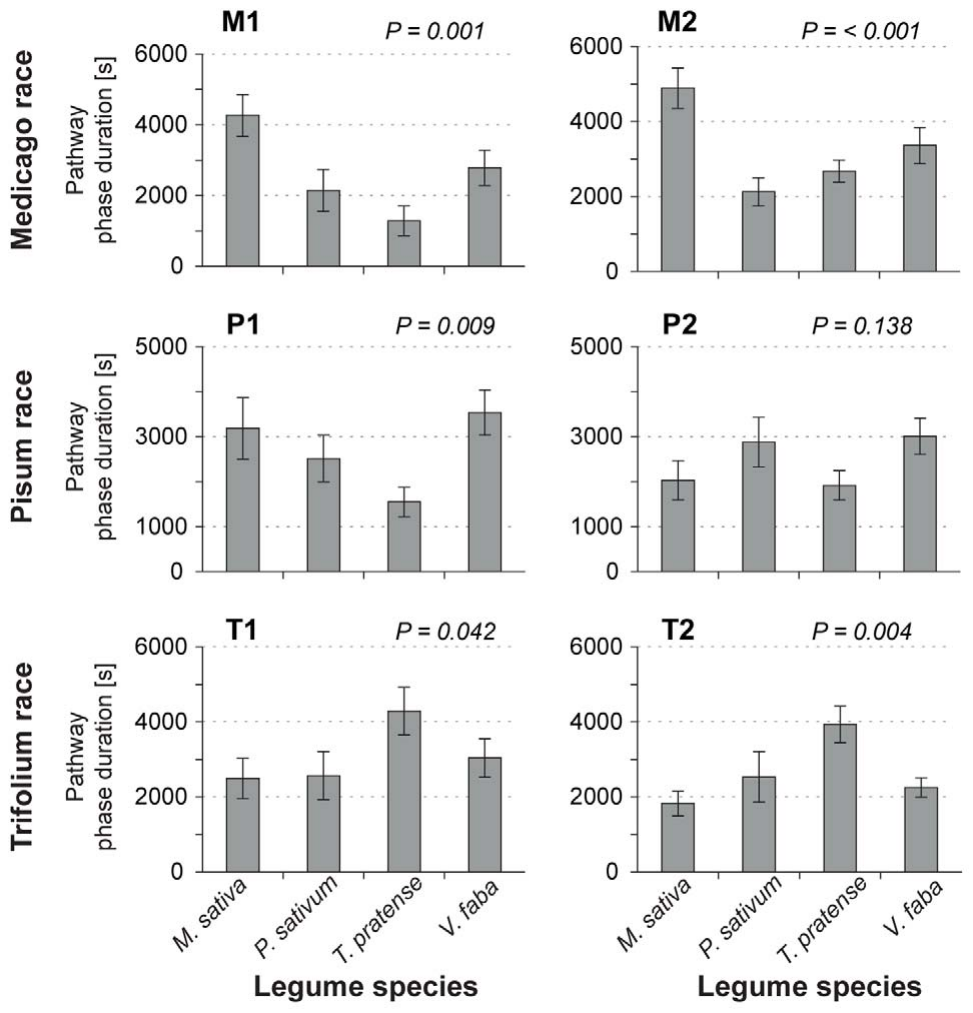
Mean duration of pathway phase during 4 The graph shows the mean duration of pathway phase (Table 2, parameter #15) for clones (M1, M2, P1, P2, T1 and T2) of each pea aphid host race on the four legume species. Bars represent mean duration +/− Std. Error. *n* = 14–21. For details about test statistics, see Table S3.

The influence of the legume species on the selected parameters was analyzed separately for each of the six aphid clones using appropriate statistical tests, i.e. parameters #1, 25, 36, 39, 41: test for equality of proportions; parameters #1, 2, 15: ANOVA; parameters #5, 8: generalized linear models with poisson/quasipoisson error structure; parameter #6: generalized linear models with quasibinomial error structure. *P*-values below significance level (<0.05) are printed in bold letters. Mean values and standard errors (or proportion data expressed as percentages, respectively) are shown in Figures 3–5, Figure 7. For details about test statistics and corresponding values, see Table S3.

**Table 3:** Test statistics for comparing the proportions of repetitive SE puncturing periods without and with subsequent feeding period and the number of feeding periods without and with preceding repetitive SE puncture periods.

	Medicago race		Pisum race		Trifolium race	
	M1	M2	P1	P2	T1	T2
Repetitive SE punctures without vs.repetitive SE punctures with subsequent feeding	**<0.001**	**<0.001**	0.273	**0.007**	**<0.001**	0.331
Feeding periods without vs. feeding periods with preceding repetitive SE punctures	–	–	**0.027**	**0.024**	**<0.001**	0.256

For details about test statistics and corresponding values, see Table S2.

#### Volatile and plant surface-related parameters

For most aphid clones the plant species did not influence the time from the start of experiment until first plant penetration (Table 2, #3), which ranged between 1000 and 6000 s. However, clones M2 and P1 took about 500–1000 s from the start of the experiment until first penetration on the universal host plant. This was significantly shorter (two to four times) than on the less suitable plant *T. pratense* (M2), and on all other plants (P1) (Table 2, #3).

#### Epidermis and mesophyll-related parameters

Aphids sometimes penetrate the plant tissue only briefly with very short probes of <30 sec. During this behavior only epidermal cells are likely to be punctured. Trifolium clones made significantly more (two-fold) very short probes on less-suitable and non-host plants than on native and universal host plants (Figure 4 A; Table 2, #5). The same effect was also observed in Medicago clone M2 (Table 2, #5).

For the Trifolium clones, we tested whether the very short probes on different plant species involve intracellular punctures. When both Trifolium clones fed upon *M. sativa*, *T. pratense* and *V. faba* most very short probes indeed contained intracellular punctures, whereas on *P. sativum* the proportion of very short probes without an intracellular puncture was significantly higher (Figure 4 B; Table 2, #6).

During probes longer than 30 s but shorter than 3 min, aphids very likely penetrate not only epidermal, but also upper mesophyll cells [21], [36]. In this parameter we could not detect significant differences among any aphid clone-plant combinations (Table 2, #8).

The pathway phase (Figure 1 B) is characterized by sheath salivation, cell puncturing and stylet bundle movement towards the sieve elements (SEs). It excludes xylem phase, penetration difficulty periods, and all SE-related phases. Aphids on their native and universal host plants spent two times longer in the pathway phase than they did on less suitable and non-host plants (Figure 5). This effect was significant for all clones except for P2 (Table 2, #15).

#### Sieve element-related parameters: *repetitive SE punctures*

**Figure 6:**
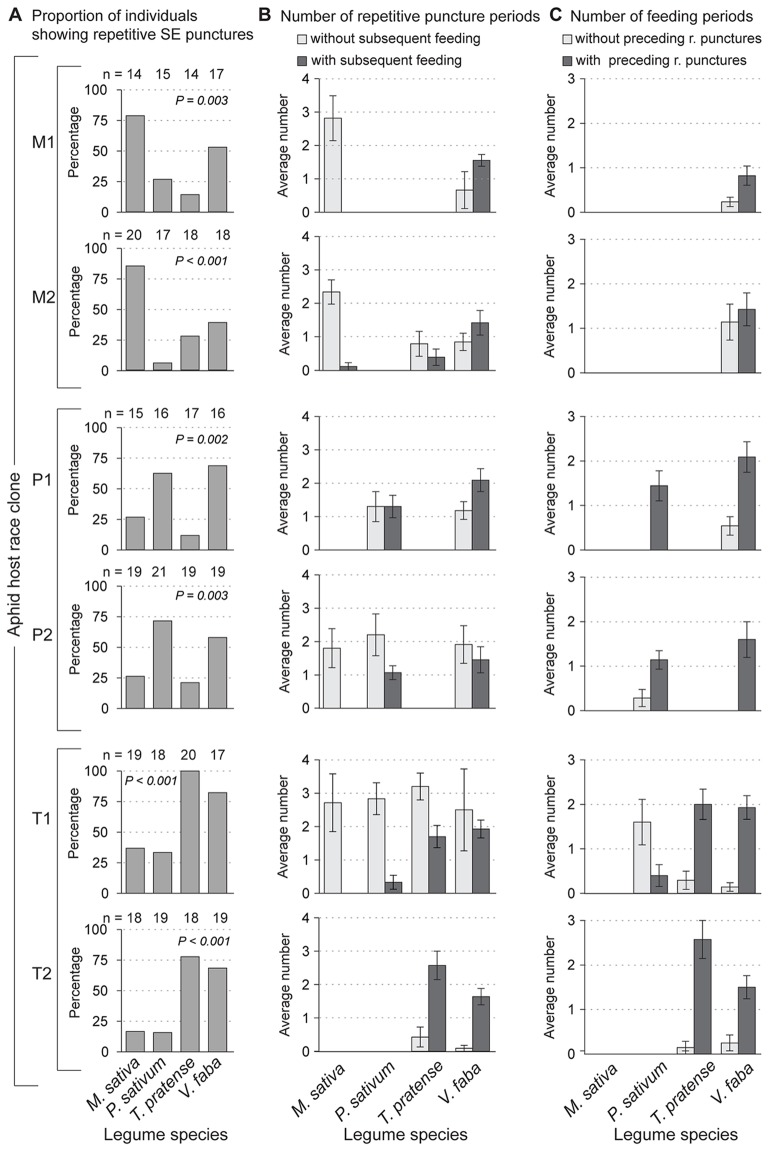
Co-occurrence of repetitive SE punctures and SE feeding. The graph shows the occurrence of repetitive SE punctures and SE feeding for clones (M1, M2, P1, P2, T1 and T2) of each pea aphid host race on the four legume species. A) Percentage of individuals showing repetitive SE punctures throughout the entire experiment (Table 2, parameter #41). B) Mean number +/− std. error of repetitive SE puncture periods per aphid with and without subsequent feeding periods (Table 3). C) Mean number +/− std. error of feeding periods per aphid with and without preceding repetitive SE puncture periods (Table 3). Missing bars in (B) and (C) are due to low replicate number caused by very low observation frequency (< five individuals showing repetitive SE punctures or SE feeding).

During repetitive SE punctures the aphid inserts its stylet repeatedly into the intracellular lumen of the SE. Approximately 40–100% of aphid individuals on the native and universal host plants showed repetitive SE puncture periods during the experiment. On less suitable and non-host plants, these proportions were significantly lower (10–30%; Figure 6 A, Table 2, #41). Successful SE feeding periods often co-occur with repetitive SE puncture periods. On native and universal host plants, most or sometimes all (clone P1 on the universal host and clone P2 on the native host plant) feeding periods followed a period of repetitive SE punctures (Figure 6 C; Table 3). However, the clones from the Medicago race which fed exclusively on the universal host but not on their native host plant, repetitively punctured SEs without subsequent feeding (Figure 6 B; Table 3). For all other clones, repetitive SE puncture periods without subsequent feeding were also observed (Figure 6 B; Table 3). The number of these events was higher compared to the number of repetitive SE puncturing periods with subsequent feeding especially on non-host plants.

#### Sieve element-related parameters: *SE salivation and SE feeding*

**Figure 7:**
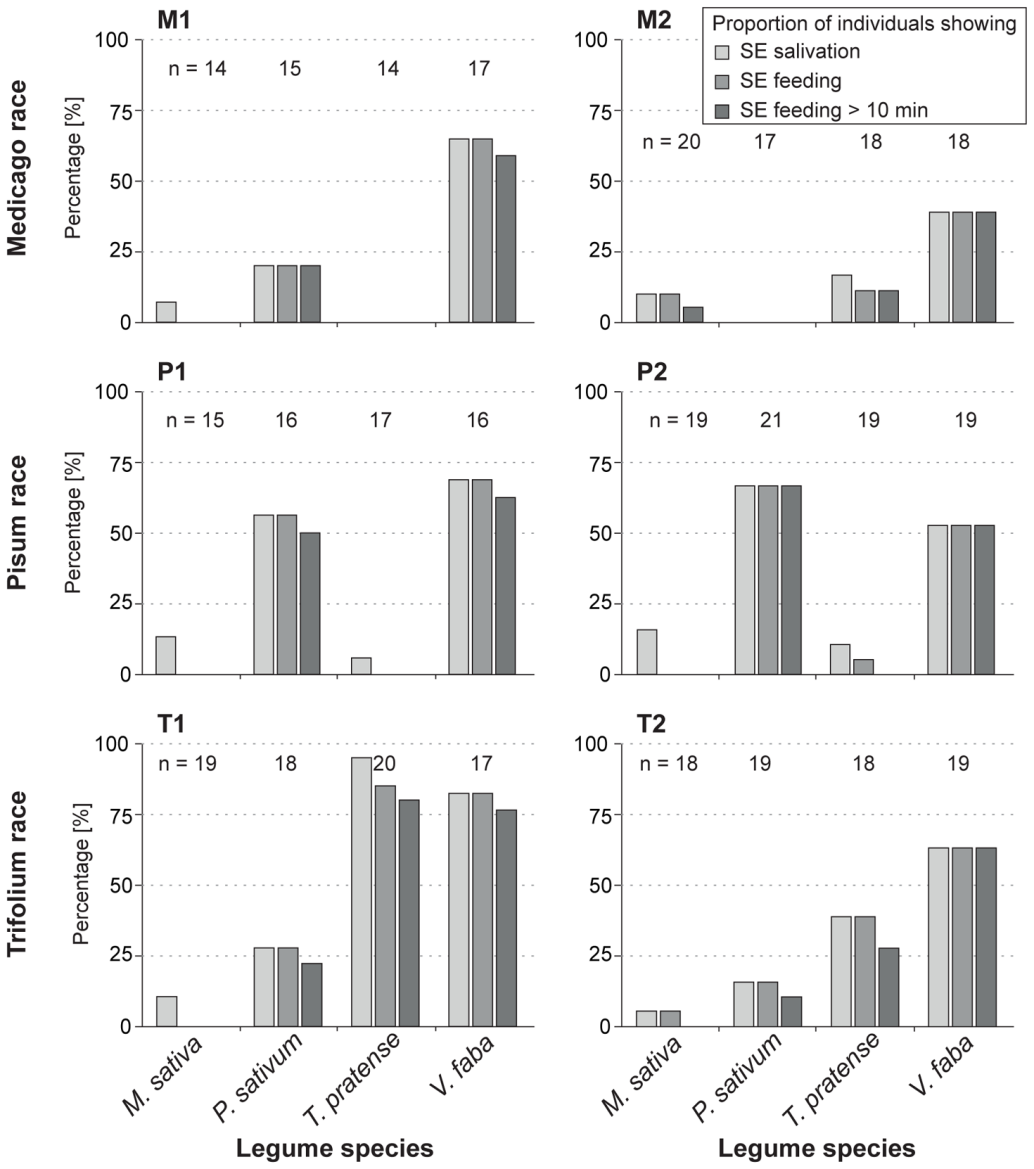
Percentage of aphid individuals showing SE related waveforms. The graph shows the percentage of individuals for clones (M1, M2, P1, P2, T1 and T2) of each pea aphid host race showing SE related waveforms on the four legume species. The influence of the plant species on proportion of individuals showing SE salivation, SE feeding and SE feeding >10 min was analyzed separately for each parameter. *P*-values all <0.001 except clone M2 (*P*<0.02) For details about test statistics, see Table 2 and Table S3.

The proportion of individuals that salivated into and/or fed on SEs during the experiment followed a uniform pattern throughout most aphid clone-plant combinations: on native and universal host plants 40–80% of individuals salivated into and/or fed on the SEs (Figure 7; Table 2, #25, 36, 39). On less suitable or non-host plants, a significantly lower proportion of individuals (0–10%) showed this behavior. Interestingly, aphid clones belonging to the Medicago race showed a pattern different from clones belonging to the other two races. On their native host plant *M. sativa*, only 0–10% of individuals of clones M1 and M2 salivated into and/or fed on SEs during the experiment, which is more similar to the behavior of other host races on the less suitable or non-host plants. On the universal host plant *V. faba*, 40–60% of the Medicago clone individuals showed SE salivation and SE feeding.

## Discussion

### Performance Differences

The clones of the three pea aphid host races tested (the Medicago, Pisum and Trifolium races) had similar patterns of performance with higher survival and greater weight on their native and the universal host *V. faba* than on non-hosts (Figure 2). Certain clones showed an intermediate performance on some legume species (Figure 2), indicating that these were less-suitable than the native and universal hosts. Performance was well-correlated with a clone’s ability to establish feeding on the sieve elements (SEs) of each plant (Figure 7). On non-host plants, few individuals (0–10%) of each clone were able to establish sustained SE feeding during the 4 hour EPG recording times, whilst on native and universal host plants many individuals (40–80%) established feeding. These results are consistent with the findings of Caillaud and Via [22] who found that Medicago and Trifolium clones established feeding on their native host, but not on a non-host plant. In our experiments, we observed a remarkable phenomenon for the Medicago clones. Both performed well on their native host plant *M. sativa* but were not able to establish SE feeding during the 4 h experiment. A similar observation was made for other clones on less suitable plants, such as P2 on *T. pratense*, which were rarely able to establish SE feeding. These inconsistencies between feeding and EPG performance might not been seen in longer EPG recordings if some clones need more time to repress plant defense responses and subsequently establish SE feeding. Another explanation might be the different age of the aphids used in the two experiments. Aphids at the start of the performance experiment were recently born, while those used in the EPG experiment were young adults (age∼10 days). First instar larvae might have a greater ability to adapt to less suitable plants, whereas young adults might have lost this ability, as shown for lepidopteran larvae [37]. In general, an intermediate number of individuals were able to establish feeding on less suitable plants (clone M2 on *T. pratense* about 12.5%, clone T1 on *P. sativum* about 25%) (Figure 7). Most pea aphid clones did not feed on non-host plants during the EPG experiment, and their low survival on these plants might be a consequence of starvation. However, the Trifolium clones on the less suitable plant *P. sativum* survived well but showed a reduced weight (Figure 2) which might be explained by various plant factors like feeding deterrents [38]–[41], low nutritional quality of the phloem sap [4], [15], or SE-located plant factors (e.g. [18], [19], [27]).

### Plant Factors Influencing Aphid Feeding

#### Volatile and surface factors

Plant volatiles or surface factors did not play an important role in host race choice on these legumes since most aphids started probing during the EPG recording regardless of clone or plant (Table 2, Parameter #1). This result confirms the findings of Caillaud [24] that there is a necessity for the pea aphid to taste the plant to discriminate among potential hosts. However, plant volatiles or surface factors could influence the time an aphid takes from being placed on the plant until first probe. In two clones, this parameter (Table 2, #3) differed significantly depending on the experimental plant. Clones M2 and P1 needed more time until beginning probing on less suitable plants compared to the native and universal hosts. Attractive substances may shorten the time to first probe on native and universal host plants, as reported for *V. faba* volatiles that had an attractive effect on *Aphis fabae* [10]. On the other hand, the lack of attractive stimuli or the presence of repellent stimuli may delay probing by *Aphis fabae* reacting to epicuticular lipids of the non-host plant *Avena sativa* [12]. The effect of volatiles or surface factors might be more widespread than can be seen in the EPG experiment since attaching wires to aphids leads to a decrease of the behavioral differences related to stylet penetration and feeding on host and non-host plants especially in the first 30 min of the aphid-plant interaction [24], [42].

#### Epidermal factors

Stylet penetration speed has been assumed to occur at a rate of approximately 0.5 cell layers per minute through the plant tissue [21], [36]. Thus, probes shorter than 30 sec should reflect factors in the epidermis. Both Trifolium clones showed a significantly higher number of these short probes on less suitable and non-host plants compared to the number on the native and universal host plant (Figure 4 A). Notably, most of the very short probes involved an intracellular puncture (Figure 4 B). Hence, intracellular epidermal factors can be assumed to be important for plant recognition and discrimination in the Trifolium clones. This result is supported by previous studies implying that factors located in peripheral plant tissue stimulate further stylet penetration of pea aphids on their native host plants [22]. Early plant recognition with subsequent rejection of less suitable or non-host plants might be one reason for the low survival or weight of the Trifolium race on these legume species during the performance experiment. In nature, early plant recognition might be advantageous since aphids with this ability might not spend as long on unsuitable plants. However, there were no hints for such behavior in the other aphid clones.

#### Mesophyll factors

After the epidermis, the next plant tissue contacted by the aphid stylets is the upper mesophyll which is probably reached between 30 sec and 3 min after plant probing starts. However, since there were no differences in the number of probes longer than 30 sec, but shorter than 3 min in any aphid clone-plant combination, factors responsible for host choice are likely not associated with the upper mesophyll tissue. The stimulating plant factors located in peripheral plant tissue proposed by Caillaud and Via [22] and Del Campo et al. [25], are likely to be located in the epidermal tissue, since the authors did not distinguish between epidermal and mesophyll located factors.

From the upper mesophyll, the aphid navigates its stylets through the plant apoplast towards the SEs. This phase of stylet penetration, known as the pathway phase, was significantly longer for most clones on their native and the universal host plant than on other plants, possibly due to plant factors stimulating further probing in native and universal host plants. These factors may be located intracellularly since during the pathway phase the aphid punctures nearly every cell it contacts [43]. Whilst puncturing cells, aphids ingest cell content and inject watery saliva [44]. Puncturing cells might serve as orientation towards the SEs [20]. Alternatively, injection of watery saliva into the cell lumen upon puncturing might condition a plant for feeding since aphid saliva is known to harbor numerous effector molecules [45]. These effector molecules interact with plant *R*-gene products or other compounds of the plant defense system [29].

#### Sieve element factors

When pea aphids reach the SEs, they often repetitively puncture these cells [46], [47]. Just as during intracellular punctures of mesophyll cells, aphids salivate into SEs and ingest at least some cell content, most likely to identify it as nutrition source [48], [49]. Individuals of every pea aphid clone reached the SEs and carried out this behavior on every species of legume tested (Figure 6A). Thus repetitive SE punctures seem to be a conserved behavior that all pea aphid clones share [19], [22]. However, the percentage of individuals reaching this step on less suitable or non-host plants was significantly lower (10–25%) than on native and universal hosts (65–100%). Thus, there must be a factor earlier in the penetration process, e.g. the lack of a stimulant factor in less suitable or non-host plants, which diminishes further probing towards the SEs. The high percentage of aphids that repetitively punctured SEs on their native or universal host plant indicates that this behavior is linked to the pea aphid’s ability to feed on a plant as proposed previously [19], [22]. In both of these previous studies, the total duration of repetitive SE puncture periods was considered rather than the number of aphids showing repetitive SE puncture periods. This total duration was shorter for aphids on non-hosts. In the present study, the analysis of repetitive SE puncture periods showed the same trend. However, sometimes it was not possible to compare the durations of repetitive SE puncture periods on host and non-host plants due to the low replicate number (<five) of repetitive SE puncture periods on non-host plants.

To find out whether repetitive SE punctures are linked to feeding on the various legume species, the number of repetitive SE puncture periods that ended up with feeding was compared to the number that ended without feeding (Figure 6 B). On less suitable or non-host plants, repetitive SE puncture periods often ended without subsequent feeding (clones P2 and T1 on less suitable plants or the non-host *M. sativa*). This behavior might reflect sampling of SE elements that were subsequently rejected for a variety of reasons, due to imbalanced amino acid composition [4], or the presence of active defense mechanisms that shut down the flow of phloem sap [50]. One mechanism to shut down phloem flow in legumes is the activation of proteins called forisomes which can block sieve plates [18], [51]. However, on native and universal host plants not every repetitive SE puncture period ended with feeding. This implies that not every SE is suitable for feeding, and multiple SEs are sampled before feeding is established. The Medicago race seems to be an extreme example of this. For both clones studied, all repetitive SE puncture periods on their native host plant ended without feeding. The fact that Medicago clones are not able to establish SE feeding on their native host plant while being able to reach and repetitively puncture SEs, points to a factor located in the SE that prevents feeding. This non-compatibility could be due to a lack of sufficient time to overcome plant defense during the 4 h EPG experiment. Alternatively, non-compatibility of the Medicago race on its native host plant might result from the lack of experience with this species as all clones had been reared on the universal host *V. faba* rather than their native host plants. However, the other host races were able to overcome lack of experience on their native hosts and establish feeding.

The occurrence of SE feeding mainly on native and universal host plants raises the question of whether repetitive puncturing of SE is a prerequisite for feeding. On universal and native hosts, SE feeding preceded by repetitive SE puncturing occurred significantly more frequently than feeding without puncturing preceding it (Figure 6 C). In some cases there was no single feeding event without repetitive SE punctures (P1 on native host plant, P2 on universal host plant). The same pattern was found for *Brevicoryne brassicae* on its host plant *Sinapis alba* where most feeding periods were preceded by repetitive SE puncture periods [47]. This pattern implies that repetitive SE punctures might fulfill a role in conditioning SEs for subsequent feeding. Salivation into SEs might also play an important role in overcoming the defense mechanisms of plants during repetitive SE puncturing. Salivary compounds can for instance suppress calcium influx into the SE right before and during SE feeding and therefore suppress SE occlusion [18]. In addition, when a salivary protein important for pea aphid survival on *V. faba* was knocked down by RNAi, ingestion from SEs was limited [30]. Notably, there is evidence from a recent study that aphid salivary effectors influence aphid performance in a plant-specific way [52]. However, so far it is not possible to answer the intriguing question of the function of repetitive SE punctures in pea aphid-legume interactions.

In general we can conclude that SE-based factors are critical in whether or not pea aphids can establish SE feeding. Their role as an ultimate barrier to feeding for certain aphid clone-legume species combinations may have driven selection for aphids to discriminate soon after initiation of penetration, which has resulted in the recognition of factors in the epidermis and the mesophyll that stimulate or deter continuation of probing towards the SEs. The observed continuum of pea aphid race ability to establish feeding on different legume species, which ranged from good to intermediate to poor, was mirrored in the performance of the different races on these same plants. This connection between host selection behavior and physiology provides a strong basis for pea aphid speciation.

## Supporting Information

Table S1 **Parameters derived from EPG recordings used to indicate the location of plant factors affecting pea aphid penetration and feeding.** For detailed information about EPG waveform standard terms and corresponding aphid behavioral correlates, please refer to Tjallingii and Esch [43], Tjallingii and Gabrys [47], and Tjallingii [53]. Additional abbreviations in column “EPG waveform”: r-pdsg = single repetitive potential drop period, E1sg = single sieve element salivation period, E1fr = fraction SE salivation period (SE salivation associated with SE feeding period).
(PDF)

Table S2 **Test statistics for comparing the proportions of repetitive SE puncture periods without and with subsequent feeding period and the proportion of feeding periods without and with preceding repetitive SE puncture periods.** KW = Kruskal-Wallis test; GLM B = Generalized linear model with binomial error structure (P-values calculated by χ2 -test, deviance values printed in regular letters); GLM Q = Generalized linear model with quasibinomial error structure (P-values calculated by F-test, F-values printed in italic letters).
(PDF)

Table S3 **Test statistics of all EPG parameters.** Statistical tests: ANOVA = Analysis of variance; KW = Kruskal-Wallis test; GLM QB = generalized linear model with quasibinomial error structure; GLM QP = generalized linear model with quasipoisson error structure; GLM P = generalized linear model with poisson error structure; EQP = test for equality of proportions. Transformations: (−) no transformation; log = logarithmic; sqrt = square root; 1/y = reciprocal transformation; asinsqrt = arcsine square root transformation. χ2 and F-values: χ2 printed in regular letters, F-values printed in italic letters. P-values: P-values <0.05 are printed in bold letters. Further signs/abbreviations: (−) = not analyzed as replicate number<five or no contrasts available. no = parameter not observed. na = parameter not analyzed.
(PDF)
