# Supplementary material for: To Feed or Not to Feed: Plant Factors Located in the Epidermis, Mesophyll, and Sieve Elements Influence Pea Aphid’s Ability to Feed on Legume Species
Source: PLoS One. 2013 Sep 30;8(9):e75298. doi: 10.1371/journal.pone.0075298 (PMC3787088; doi:10.1371/journal.pone.0075298)
Supplement: Table S1 — Parameters derived from EPG recordings used to indicate the location of plant factors affecting pea aphid penetration and feeding. For detailed information about EPG waveform standard terms and corresponding aphid behavioral correlates, please refer to Tjallingii and Esch [43], Tjallingii and Gabrys [47], and Tjallingii [53]. Additional abbreviations in column “EPG waveform”: r-pdsg = single repetitive potential drop period, E1sg = single sieve element salivation period, E1fr = fraction SE salivation period (SE salivation associated with SE feeding period). (PDF) [file pone.0075298.s001.pdf]

Tissue	#	EPG parameter	EPG waveform
Global	1	Proportion of individuals starting penetration	Pathway
	2	Total penetration time	All
Volatile/Surface	3	Time from start of experiment to first probe	All
Surface/Epidermis/Mesophyll	4	First probe duration	All
Epidermis	5	Number of probes shorter than 30 sec	Pathway
	6	Proportion of probes shorter than 30 sec with cell puncture and without cell puncture	Pathway/pd
	7	Number of probes shorter than 30 sec before first SE salivation	Pathway
Epidermis/Mesophyll	8	Number of probes shorter than 3 min	Pathway
	9	Number of probes shorter than 3 min before first SE salivation	Pathway
	10	Number of probes	All
	11	Number of occurrences of probes before first SE salivation or feeding	All
Stress Mesophyll	12	Proportion of individuals showing penetration difficulties	F
	13	Number of penetration difficulties	F
	14	Total duration of penetration difficulties	F
Mesophyll	15	Total duration of pathway phase	Pathway
	16	Cell puncture frequency per min pathway phase	Pathway/pd
	17	Proportion of individuals showing unknown waveform	Unknown waveform
	18	Number of unknown waveforms	Unknown waveform
	19	Total duration of unknown waveform	Unknown waveform
Mesophyll/SEs	20	Time from first probe to first SE salivation	All but E
	21	Time from first probe to first SE feeding	All but E2
	22	Time from first probe to first sustained SE feeding	All but E2 > 10 min
	23	Time from start of successful probe to first SE salivation	All but np, E2
	24	Time from experiment start to first sustained SE feeding	All but E1, E2 < 10 min
SEs	25	Proportion of individuals showing SE salivation	E1
	26	Number of SE salivations	E1
	27	Total duration of SE salivation	E1
	28	Proportion of individuals showing SE salivation without subsequent feeding	E1sg
	29	Number of SE salivations without subsequent feeding	E1sg
	30	Total duration of SE salivations without subsequent feeding	E1sg
	31	Number of SE salivations with subsequent feeding	E1fr
	32	Total duration of SE salivation with subsequent feeding	E1fr
	33	Contribution of SE salivation to SE phase	E1/E2
	34	Duration of first SE salivation with subsequent feeding	E1/E2
	35	Total duration of SE salivation with subsequent feeding	E1/E2
	36	Proportion of individuals showing SE feeding	E2
	37	Number of SE feedings	E2
	38	Total duration of SE feeding	E2
	39	Proportion of individuals showing sustained SE feeding	E2 > 10 min
	40	Number of sustained SE feedings	E2 > 10 min
	41	Proportion of individuals showing repetitive SE punctures	r-pd
	42	Proportion of individuals showing single repetitive SE punctures	r-pdsg
	43	Proportion of individuals showing repetitive SE punctures with subsequent SE salivation	r-pdE1
	44	Proportion of individuals showing repetitive SE punctures with subsequent SE salivation/feeding	r-pdE1E2
	45	Proportion of individuals showing repetitive SE punctures with subsequent SE salivation/sustainedfeeding	r-pdE1E2 > 10 min
	46	Number of repetitive SE puncture periods	r-pd
	47	Total duration of repetitive SE puncture periods	r-pd
	48	Number of single repetitive SE puncture periods	r-pdsg
	49	Number of repetitive SE puncture periods with subsequent SE salivation	r-pdE1
	50	Number of repetitive SE puncture periods with subsequent SE salivation/feeding	r-pdE1E2
	51	Number of repetitive SE puncture periods with subsequent SE salivation/sustainedfeeding	r-pdE1E2 > 10 min
Xylem	52	Proportion of individuals showing xylem ingestion	G
	53	Number of xylem ingestions	G
	54	Total duration of xylem ingestions	G
