# Supplementary material for: To Feed or Not to Feed: Plant Factors Located in the Epidermis, Mesophyll, and Sieve Elements Influence Pea Aphid’s Ability to Feed on Legume Species
Source: PLoS One. 2013 Sep 30;8(9):e75298. doi: 10.1371/journal.pone.0075298 (PMC3787088; doi:10.1371/journal.pone.0075298)
Supplement: Table S2 — Test statistics for comparing the proportions of repetitive SE puncture periods without and with subsequent feeding period and the proportion of feeding periods without and with preceding repetitive SE puncture periods. KW = Kruskal-Wallis test; GLM B = Generalized linear model with binomial error structure (P-values calculated by χ2 -test, deviance values printed in regular letters); GLM Q = Generalized linear model with quasibinomial error structure (P-values calculated by F-test, F-values printed in italic letters). (PDF) [file pone.0075298.s002.pdf]

	Medicago race						Pisum race						Trifolium race					
	M1			M2			P1			P2			T1			T2		
	Stat. test	χ^2 or F	P	Stat. test	χ^2 or F	P	Stat. test	χ^2 or F	P	Stat. test	χ^2 or F	P	Stat. test	χ^2 or F	P	Stat. test	χ^2 or F	P
Repetitve SE punctures without vs. repetitive SE punctures with subsequent feeding	KW	17.846	< **0.001**	KW	14.327	< **0.001**	GLM Q	*1.275*	0.273	KW	9.790	**0.007**	KW	21.375	< **0.001**	GLM B	-0.946	0.331
Feeding without vs. feeding with preceding repetitive SE punctures	-	-	-	-	-	-	GLM B	-4.881	**0.027**	GLM B	-5.099	**0.024**	GLM B	-21.936	< **0.001**	GLM B	-1.293	0.256

TABLE S2: Test statistics for comparing the proportions of repetitive SE puncture periods without and with subsequent feeding period and the proportion of feeding periods without and with preceding repetitive SE puncture periods. KW = Kruskal-Wallis test; GLM B = Generalized linear model with binomial errors (*P*-values calculated by χ^2 -test, deviance values printed in regular letters); GLM Q = Generalized linear model with quasibinomial errors (*P*-values calculated by *F*-test, *F*-values printed in italic letters).
