# Supplementary material for: To Feed or Not to Feed: Plant Factors Located in the Epidermis, Mesophyll, and Sieve Elements Influence Pea Aphid’s Ability to Feed on Legume Species
Source: PLoS One. 2013 Sep 30;8(9):e75298. doi: 10.1371/journal.pone.0075298 (PMC3787088; doi:10.1371/journal.pone.0075298)
Supplement: Table S3 — Test statistics of all EPG parameters. Statistical tests: ANOVA = Analysis of variance; KW = Kruskal-Wallis test; GLM QB = generalized linear model with quasibinomial error structure; GLM QP = generalized linear model with quasipoisson error structure; GLM P = generalized linear model with poisson error structure; EQP = test for equality of proportions. Transformations: (−) no transformation; log = logarithmic; sqrt = square root; 1/y = reciprocal transformation; asinsqrt = arcsine square root transformation. χ2 and F-values: χ2 printed in regular letters, F-values printed in italic letters. P-values: P-values <0.05 are printed in bold letters. Further signs/abbreviations: (−) = not analyzed as replicate number<five or no contrasts available. no = parameter not observed. na = parameter not analyzed. (PDF) [file pone.0075298.s003.pdf]

		EPG parameter	M1				M2				P1			
			Stat. test	Transf.	χ^2 or *F*	*P*	Stat. test	Transf.	χ^2 or *F*	*P*	Stat. test	Transf.	χ^2 or *F*	*P*
Global	1	Proportion of individuals starting penetration	EQP	-	2.292	0.514	EQP	-	1.158	0.763	EQP	-	0.246	0.970
	2	Total penetration time	AOV	-	*2.439*	0.074	KW	-	6.038	0.110	AOV	-	*7.162*	**<0.001**
Volatile/Surface	3	Time from start of experiment to first probe	KW	-	1.382	0.710	AOV	log	*3.312*	**0.025**	KW	-	8.910	**0.031**
Surface/Epidermis/Mesophyll	4	First probe duration	KW	-	6.029	0.110	AOV	log	*2.587*	0.060	KW	-	1.886	0.596
Epidermis	5	Number of probes shorter than 30 sec	GLM QP	-	*0.538*	0.659	GLM QP	-	*3.586*	**0.021**	GLM QP	-	*0.550*	0.651
	6	Proportion of probes shorter than 30 sec without/with pd	na	-	-	-	na	-	-	-	na	-	-	-
	7	Number of probes shorter than 30 sec before first E1	-	-	-	-	-	-	-	-	GLM QP	-	*< 0.001*	0.990
Epidermis/Mesophyll	8	Number of probes shorter than 3 min	GLM QP	-	*1.048*	0.389	GLM QP	-	*1.748*	0.171	GLM QP	-	*1.795*	0.162
	9	Number of probes shorter than 3 min before first E1	-	-	-	-	-	-	-	-	-	-	-	-
	10	Number of probes	GLM QP	-	*0.589*	0.625	GLM QP	-	*0.160*	0.923	GLM QP	-	*2.039*	0.118
	11	Number of occurrences of probes before first E	-	-	-	-	-	-	-	-	GLM QP	-	*0.583*	0.455
Stress Mesophyll	12	Proportion of individuals showing F	EQP	-	4.509	0.212	EQP	-	3.332	0.343	EQP	-	4.070	0.254
	13	Number of F	AOV	-	*1.072*	0.369	KW	-	2.947	0.400	AOV	-	*0.019*	0.893
	14	Total duration of F	KW	-	1.746	0.627	GLM P	-	15.923	0.546	GLM P	-	7.489	0.069
Mesophyll	15	Total duration of pathway phase	AOV	sqrt	*5.874*	**0.001**	AOV	sqrt	*7.280*	**<0.001**	AOV	sqrt	*4.243*	**0.009**
	16	Potential drop frequency per min pathway phase	KW	-	4.440	0.218	AOV	log	*2.422*	0.073	AOV	-	*1.740*	0.169
	17	Proportion of individuals showing unknown waveform	EQP	-	5.017	0.171	EQP	-	11.692	**0.009**	EQP	-	6.403	0.094
	18	Number of unknown waveforms	KW	-	5.017	0.398	KW	-	6.229	0.313	GLM QP	-	*1.203*	0.318
	19	Total duration of unknown waveform	AOV	log	*0.138*	0.872	AOV	log	*1.914*	0.165	AOV	log	*0.831*	0.448
Mesophyll/SEs	20	Time from first probe to first E1	-	-	-	-	-	-	-	-	KW	-	0.013	0.909
	21	Time from first probe to first E2	-	-	-	-	-	-	-	-	KW	-	0.071	0.790
	22	Time from first probe to first E2 > 10 min	-	-	-	-	-	-	-	-	KW	-	0.387	0.534
	23	Time from start of successful probe to first E1	-	-	-	-	-	-	-	-	AOV	log	*1.196*	0.289
	24	Time from experiment start to first E2 > 10 min	-	-	-	-	-	-	-	-	KW	-	0.640	0.424
SEs	25	Proportion of individuals showing E1	EQP	-	21.542	**<0.001**	EQP	-	10.553	**0.014**	EQP	-	20.349	**<0.001**
	26	Number of E1	-	-	-	-	-	-	-	-	GLM P	-	9.310	**0.009**
	27	Total duration of E1	-	-	-	-	-	-	-	-	AOV	1/y	*0.960*	0.340
	28	Proportion of individuals showing single E1	EQP		0.988	0.804	EQP	-	1.905	0.592	EQP	-	0.889	0.828
	29	Number of single E1	-	-	-	-	-	-	-	-	-	-	-	-
	30	Total duration of single E1	-	-	-	-	-	-	-	-	-	-	-	-
	31	Number of SE salivation fractions	-	-	-	-	-	-	-	-	GLM P	-	13.866	0.096
	32	Total duration of SE salivation fractions	-	-	-	-	-	-	-	-	AOV	log	*0.003*	0.954
	33	Contribution of SE salivation to SE phase	-	-	-	-	-	-	-	-	AOV	asinsqrt	*0.189*	0.669
	34	Total duration of first E1->E2	-	-	-	-	-	-	-	-	KW	-	1.052	0.305
	35	Total duration E1->E2	-	-	-	-	-	-	-	-	AOV	-	*0.071*	0.793
	36	Proportion of individuals showing E2	EQP	-	24.881	**<0.001**	EQP	-	11.618	**0.009**	EQP	-	29.673	**<0.001**
	37	Number of E2	-	-	-	-	-	-	-	-	GLM P	-	12.857	0.063
	38	Total duration of E2	-	-	-	-	-	-	-	-	AOV	-	*0.076*	0.786
	39	Proportion of individuals showing sustained E2	EQP	-	21.598	**<0.001**	EQP	-	13.742	**0.003**	EQP	-	25.662	**<0.001**
	40	Number of sustained E2	-	-	-	-	-	-	-	-	GLM P	-	4.112	0.308
	41	Proportion of individuals showing r-pd	EQP	-	14.226	**0.003**	EQP	-	25.989	**<0.001**	EQP	-	15.268	**0.002**
	42	Proportion of individuals showing single r-pd	EQP	-	20.780	**<0.001**	EQP		31.126	**<0.001**	EQP	-	7.819	**0.050**
	43	Proportion of individuals showing r-pd->E1	EQP	-	1.973	0.578	EQP	-	1.905	0.592	EQP	-	2.204	0.531
	44	Proportion of individuals showing r-pd->E1->E2	EQP	-	4.967	0.174	EQP	-	9.560	**0.023**	EQP	-	12.506	**0.006**
	45	Proportion of individuals showing r-pd->E1->E2 sustained	EQP	-	22.786	**<0.001**	EQP	-	10.756	**0.013**	EQP	-	25.662	**<0.001**
	46	Number of r-pd	GLM QP	-	*0.252*	0.622	GLM P	-	25.232	0.335	GLM P	-	15.201	0.273
	47	Total duration of r-pd	KW	-	4.053	**0.044**	KW	-	0.315	0.854	AOV	sqrt	*1.642*	0.216
	48	Number of single r-pd	-	-	-	-	KW	-	3.426	0.635	GLM P	-	5.700	0.303
	49	Number r-pd->E1	-	-	-	-	-	-	-	-	-	-	-	-
	50	Number r-pd->E1->E2	-	-	-	-	-	-	-	-	-	-	-	-
	51	Number r-pd->E1->E2 sustained	-	-	-	-	-	-	-	-	KW	-	1.063	0.588
Xylem	52	Proportion of individuals showing xylem ingestion	EQP	-	21.676	**<0.001**	EQP	-	-	0.342	no	-	-	-
	53	Number of xylem ingestions	-	-	-	-	-	-	-	-	no	-	-	-
	54	Total duration of xylem ingestions	-	-	-	-	-	-	-	-	no	-	-	-

		EPG parameter	P2				T1				T2			
			Stat. test	Transf.	χ^2 or F	P	Stat. test	Transf.	χ^2 or F	P	Stat. test	Transf.	χ^2 or F	P
Global	1	Proportion of individuals starting penetration	EQP	-	1.198	0.754	EQP	-	2.264	0.520	EQP	-	0.248	0.970
	2	Total penetration time	AOV	sqrt	*6.747*	**<0.001**	KW	-	17.289	**<0.001**	KW	-	7.023	0.071
Volatile/Surface	3	Time from start of experiment to first probe	KW	-	5.067	0.167	KW	-	5.394	0.145	KW	-	2.358	0.501
Surface/Epidermis/Mesophyll	4	First probe duration	KW	-	5.554	0.135	KW	-	15.367	**0.002**	KW	-	3.833	0.280
Epidermis	5	Number of probes shorter than 30 sec	GLM QP	-	*1.315*	0.278	GLM QP	-	*3.662*	**0.017**	GLM QP	-	*4.716*	**0.005**
	6	Proportion of probes shorter than 30 sec without/with pd	na	-	-	-	GLM QB	-	*7.4434*	**0.008**	GLM QB	-	*17.004*	**<0.001**
	7	Number of probes shorter than 30 sec before first E1	-	-	-	-	GLM QP	-	*0.990*	0.384	GLM QP	-	*2.498*	0.132
Epidermis/Mesophyll	8	Number of probes shorter than 3 min	GLM QP	-	*1.525*	0.218	GLM QP	-	*0.689*	0.563	GLM QP	-	*1.307*	0.283
	9	Number of probes shorter than 3 min before first E1	GLM QP	-	*1.816*	0.199	GLM QP	-	*0.335*	0.569	-	-	-	-
	10	Number of probes	GLM QP	-	*0.872*	0.460	GLM QP	-	*1.285*	0.286	GLM QP	-	*1.695*	0.176
	11	Number of occurrences of probes before first E	GLM QP	-	*0.583*	0.455	GLM QP	-	*1.080*	0.351	GLM QP	-	*4.248*	0.055
Stress Mesophyll	12	Proportion of individuals showing F	EQP	-	9.293	**0.026**	EQP	-	9.473	**0.024**	EQP	-		0.156
	13	Number of F	AOV	sqrt	*0.754*	0.461	KW	-	0.388	0.533	AOV	log	*1.889*	0.152
	14	Total duration of F	GLM P	-	7.627	0.490	GLM P	-	4.438	0.714	GLM P	-	8.747	0.197
Mesophyll	15	Total duration of pathway phase	AOV	-	*1.892*	0.138	AOV	sqrt	*2.882*	**0.042**	KW	-	13.473	**0.004**
	16	Potential drop frequency per min pathway phase	AOV	-	*4.834*	**0.004**	AOV	1/y	*1.525*	0.216	AOV	-	*2.006*	0.121
	17	Proportion of individuals showing unknown waveform	EQP	-	11.277	**0.010**	EQP	-	8.432	**0.038**	EQP	-	6.291	0.098
	18	Number of unknown waveforms	KW	-	4.697	0.319	KW	-	2.190	0.701	GLM P	-	9.175	0.831
	19	Total duration of unknown waveform	KW	-	2.890	0.236	KW	-	2.539	0.281	KW	-	0.417	0.519
Mesophyll/SEs	20	Time from first probe to first E1	AOV	log	*0.472*	0.500	KW	-	1.011	0.603	KW	-	**0.007**	0.933
	21	Time from first probe to first E2	AOV	log	*0.789*	0.384	KW	-	1.613	0.446	KW	-	0.064	0.800
	22	Time from first probe to first E2 > 10 min	AOV	log	*1.106*	0.305	KW	-	0.094	0.759	KW	-	0.278	0.598
	23	Time from start of successful probe to first E1	KW	-	0.123	0.725	KW	-	3.312	0.191	KW	-	0.864	0.353
	24	Time from experiment start to first E2 > 10 min	AOV	-	*5.773*	**0.025**	KW	-	**0.048**	0.826	KW	-	**0.000**	1.000
SEs	25	Proportion of individuals showing E1	EQP	-	19.261	**<0.001**	EQP	-	38.482	**<0.001**	EQP	-	17.188	**<0.001**
	26	Number of E1	GLM P	-	21.529	0.498	GLM QP	-	*32.865*	**0.028**	GLM P	-	5.635	0.076
	27	Total duration of E1	KW	-	0.343	0.558	KW	-	7.491	**0.024**	AOV	log	-	**0.003**
	28	Proportion of individuals showing single E1	EQP	-	3.699	0.296	EQP	-	3.771	0.287	EQP	-	1.657	0.647
	29	Number of single E1	-	-	-	-	-	-	-	-	-	-	-	-
	30	Total duration of single E1	-	-	-	-	-	-	-	-	-	-	-	-
	31	Number of SE salivation fractions	GLM P	-	14.715	0.808	GLM QP	-	0.341	0.713	GLM P	-	9.106	0.169
	32	Total duration of SE salivation fractions	KW	-	0.219	0.640	AOV	log	*1.394*	0.262	AOV	log	*5.028*	**0.039**
	33	Contribution of SE salivation to SE phase	AOV	asinsqrt	*0.009*	0.287	AOV	asinsqrt	*1.489*	0.240	AOV	asinsqrt	*15.344*	**0.001**
	34	Total duration of first E1->E2	AOV	-	*2.029*	0.168	AOV	log	*2.007*	0.151	AOV	sqrt	*2.129*	0.163
	35	Total duration E1->E2	AOV	-	*1.675*	0.209	AOV	log	*0.561*	0.576	AOV	-	*3.254*	0.089
	36	Proportion of individuals showing E2	EQP	-	30.472	**<0.001**	EQP	-	39.448	**<0.001**	EQP	-	17.188	**<0.001**
	37	Number of E2	GLM P	-	10.014	0.736	GLM P	-	26.134	0.882	GLM P	-	9.106	0.169
	38	Total duration of E2	AOV	-	-	0.210	KW	-	1.375	0.503	AOV	-	*3.479*	0.080
	39	Proportion of individuals showing sustained E2	EQP	-	33.856	**<0.001**	EQP	-	36.077	**<0.001**	EQP	-	22.533	**<0.001**
	40	Number of sustained E2	GLM P	-	1.272	0.739	GLM P	-	10.060	0.542	KW	-	2.516	0.284
	41	Proportion of individuals showing r-pd	EQP	-	14.292	**0.003**	EQP	-	27.000	**<0.001**	EQP	-	24.450	**<0.001**
	42	Proportion of individuals showing single r-pd	EQP	-	6.856	0.077	EQP	-	24.965	**<0.001**	EQP	-	19.808	**<0.001**
	43	Proportion of individuals showing r-pd->E1	EQP	-	6.772	0.080	EQP	-	0.796	0.851	EQP	-	0.731	0.866
	44	Proportion of individuals showing r-pd->E1->E2	EQP	-	6.207	0.102	EQP	-	9.016	**0.029**	EQP	-	9.976	**0.019**
	45	Proportion of individuals showing r-pd->E1->E2 sustained	EQP	-	29.211	**<0.001**	EQP	-	44.117	**<0.001**	EQP	-	22.346	**<0.001**
	46	Number of r-pd	GLM QP	-	*0.974*	0.390	GLM QP	-	*0.608*	0.614	GLM QP	-	*8.917*	**0.006**
	47	Total duration of r-pd	KW	-	7.508	**0.023**	KW	-	22.620	**<0.001**	AOV	log	*0.189*	0.668
	48	Number of single r-pd	GLM QP	-	*0.451*	0.643	GLM QP	-	*0.972*	0.417	GLM P	-	17.326	**0.035**
	49	Number r-pd->E1	-	-	-	-	-	-	-	-	-	-	-	-
	50	Number r-pd->E1->E2	-	-	-	-	GLM P	-	1.881	0.466	-	-	-	-
	51	Number r-pd->E1->E2 sustained	GLM P	-	0.000	1.000	GLM P	-	10.124	0.406	GLM P	-	3.014	0.533
Xylem	52	Proportion of individuals showing xylem ingestion	EQP	-	3.996	0.262	EQP	-	1.847	0.605	no	-	-	-
	53	Number of xylem ingestions	-	-	-	-	-	-	-	-	no	-	-	-
	54	Total duration of xylem ingestions	-	-	-	-	-	-	-	-	no	-	-	-
